# Mapping principles and worked examples for structural learning: effects of content complexity

**DOI:** 10.3389/fpsyg.2023.1241873

**Published:** 2023-08-23

**Authors:** Hsinmei Liao

**Affiliations:** Department of Human Development and Psychology, Tzu Chi University, Hualien City, Taiwan

**Keywords:** principle learning, worked example, mathematics learning, content complexity, analogical comparison and mapping, relational learning, conceptual knowledge, structural knowledge

## Abstract

Drawing connections between principles and worked examples is an approach to learning and instruction, but it is poorly understood. This study investigated the effects of principle and example complexity on learners’ ability to map principles and worked examples. The complexity of a principle or example was determined based on the number of concepts and relationships involved. 138 college students were randomly assigned to one of the mapping conditions: principle–simple example, principle–complex example, simple example–simple example, simple example–complex example, and complex example–complex example. The participants studied related materials and completed a free-mapping and a guided-mapping task for a simple and a complex probability principle. The effects of the mapping activities were measured in terms of gains in structural and conceptual knowledge. For the simple principle, principle–example mapping led to fewer nonrelational comparisons (standalone concepts) than did example–example mapping and an equal number of relational comparisons (interconnected concepts). For the complex principle, principle–example mapping led to fewer nonrelational but more relational comparisons than example–example mapping did. Principle–example mapping of corresponding content was more difficult than example–example mapping was. However, principle–example mapping of noncorresponding content was as easy as or easier than example–example mapping. The two forms of mapping resulted in equivalent gains in structural and conceptual knowledge. The findings of this study expand the understanding of analogical reasoning and learning through mapping and comparison of abstract and concrete content. The findings indicate that principle–example mapping enables learners to overcome the obstacles of comprehending abstract or general information and to identify the interrelationships of the individual concepts in formal structures.

## 1. Introduction

For school subjects such as mathematics and physics, learning involves acquiring concepts and their definitions and, occasionally, symbols representing the concepts. A crucial goal of learning is the formation of relations or structures among concepts because relations may illustrate or define some principles, such as rules, laws, and theorems, or more complex phenomena. To illustrate, for any two numbers *a* and *b*, we can calculate their sum raised to the power of *n*, which can be represented by ${\left(a+b\right)}^{n}$ . For all the possible *n* values, the results can be collectively represented through the binomial theorem ${\left(a+b\right)}^{n}=\sum_{x=0}^{n}\left(\begin{array}{c} n\\ x \end{array}\right)a^{x}b^{n-x}$ . Within the theorem, the concepts of a binomial, an exponent, a sum, and a binomial expansion are linked by relations. Principles are frequently delineated in general or abstract statements to express the relations among the concepts underlying such statements. Such principles are referred to as *principled information* in the present study.

Directly tackling to-be-learned principles can be difficult because principles are abstract by nature (e.g., Chi et al., 1989; Koedinger et al., 2008). Concrete examples are often used to present situations in which a target principle can be applied or how the principle can be used to solve problems. The examples presented in the present study serve both of these functions and are considered *worked examples*. The following illustrates when the binomial theorem can be applied. Assume a random incident results in one of two outcomes A or B and the probability of A occurring is 0.2 and that of B occurring is 0.8. We can determine the probability of A happening 3 times and B happening 7 times, regardless the order of occurrence, by this part of the theorem $\left(\begin{array}{c} n\\ x \end{array}\right)a^{x}b^{n-x}$.

Understanding the associations between principled information and worked examples is also a crucial learning goal because worked examples serve as actualizations of target principles. Other forms of information, such as tables or figures, can supplement or even serve as targets of learning. Connecting various forms of information is the ultimate goal of learning because understanding involves developing a web of interconnected knowledge (e.g., Lopez et al., 2014). Not only is understanding valuable in itself, it is also valuable for later problem-solving. Developing a network of knowledge, however, can be difficult (e.g., Renkl, 1997; Dixon, 2005; Schwartz et al., 2011; Belenky and Schalk, 2014).

The focus of the present study was the connections learners form between domain principles and worked examples. Examples may be the most frequently used instructional aids, and the structural information they convey should reflect target principles. Numerous studies have investigated how worked examples can be used to teach target principles (e.g., Gerjets et al., 2006; Nokes-Malach et al., 2012), for example, by pairing the worked examples with instructional explanations. However, these studies have typically focused on how worked examples should be implemented rather than on domain principles. The current study investigated the characteristics of directly connecting principled information and related worked examples through mapping. This work would contribute to deepen the understanding of how principled information and worked examples become associated and to formulate effective instructional and learning strategies.

## 2. Connecting as analogical mapping

In analogical thinking or problem-solving, an individual uses the solution to a source problem to solve a target problem. If the source and target problems have a common conceptual structure despite belonging to different content domains, the target problem may be solved. Cognitive psychologists have elucidated the processes underlying analogical reasoning (e.g., Gick and Holyoak, 1980; Gentner, 1989). The individual first retrieves an appropriate analogue or solution to a source problem. Then, they align or map the entities and relations of the source and target problems. In next steps, the individual makes inferences regarding the target problem on the basis of their knowledge of the source problem and generalizes or transfers the solution of the source problem to the target problem.

An analogy generally involves two targets of concrete problems or situations, such as determining whether two worked examples have the same conceptual structure of a binomial distribution. The literature on analogical learning and comparison has offered a wealth of evidence regarding the effects of such learning and its incorporation into instructional strategies (e.g., Gentner et al., 2003). In the current study, the definition of analogy was expanded to include instances involving one concrete and one abstract situation, that is, involving a worked example and its underlying principle. In a learning context in which a source and a target problem are provided to a learner, mapping is likely the first step the learner takes because through mapping, the learner can determine similarities in the conceptual structures of the two problems and thereby delineate the conceptual structure shared by the problems. In light of this, a new strategy of learning by mapping or comparing an example and its underlying principle was proposed, and its potential applicability was explored.

This hybrid approach was developed with consideration of the following. First, for many learners, principles are too abstract to understand and too general to apply. By contrast, an example may illustrate only certain aspects of its underlying principle and be too specific for generalizations to be made (e.g., Reed and Bolstad, 1991). Therefore, learning by connecting the two forms of material may be more effective than learning either alone (e.g., Chi et al., 1989; Fyfe et al., 2014) because they compensate for each other’s disadvantages. Second, the conceptual structure of a principle is complete and can serve as a model for comparison. In cases in which examples or problems do not illustrate all aspects of a conceptual structure, the learner is likely to be prompted to further investigate and acquire the remaining aspects of the structure (Schwartz et al., 2011; Lachner and Nückles, 2015). Third, principle–example mapping may enable learners to focus on structural similarities between a source and a target because principles involve general entities and relations rather than distracting contextual information. These three considerations highlight the potential advantages of the proposed hybrid approach over traditional mapping approaches. Because no study on such a hybrid approach was identified in the literature, the following presents a review of empirical studies on analogical reasoning and learning.

## 3. Connecting worked examples

Analogizing concrete situations is crucial in both instruction and learning and has been researched and applied in various school subjects (Alfieri et al., 2013; Dumas et al., 2013; Richland and Simms, 2015; Goldwater and Schalk, 2016). However, simply providing a learner with a source and target problem may not lead to the desired learning outcomes. Gentner et al. (2003) discovered that comparing two examples and participating in analogical encoding (i.e., a guided comparison of two examples) both resulted in more favorable learning and transfer than did studying the two examples in order and the baseline condition, that is, the participants simply solving the problems. Without guidance, many learners do not engage in active learning processes, such as analogical comparison (Chi et al., 1989; Renkl, 1997). Therefore, learning content must be deliberately connected.

Furthermore, learners may struggle to align or map the elements of different content (e.g., Bassok, 1990; Ross and Kilbane, 1997). To ensure more favorable learning outcomes, learners must be guided through analogical mapping with instruction. Such external support can lead them to focus the comparisons on structural components and thus increase the likelihood of the learner comprehending the underlying principle. If the learner does not receive guidance, they may focus on superficial features of the problems provided that are not relevant to the learning goals (e.g., Schwartz et al., 2011; Day et al., 2015). Several studies (Gentner et al., 2003; Richland and McDonough, 2010) have discovered that comparisons conducted with guidance are generally more beneficial than comparisons conducted without and have reported the effects of improvements in solving novel problems, or procedural knowledge, and in conceptual understanding, or schematic knowledge (e.g., Cummins, 1992; Gentner et al., 2003; Rittle-Johnson and Star, 2007; Richland and McDonough, 2010; Nokes-Malach et al., 2012).

With the exception of asking learners to map or compare two isomorphic problems with the same solution (e.g., Ross and Kennedy, 1990; Novick and Holyoak, 1991; Cummins, 1992), asking learners to map or compare problems that can be solved using different solution procedures but involving the same underlying principle can empower them to more effectively learn the conceptual structure of the principle (e.g., Catrambone and Holyoak, 1990; Reed et al., 2012). Such nonisomorphic problems often have different subgoals and therefore illustrate specific aspects of the target principle. These differences cause the problems to appear different, and different steps to be required to obtain the necessary values for obtaining a solution. Research indicates that comparing such problems leads to greater success in solving isomorphic problems and awareness of the conceptual aspects reflected in the problems’ subgoals (e.g., Catrambone and Holyoak, 1990).

Some studies have obtained differential findings regarding the learning effects of comparing these types of problems (Reed, 1989; Reed et al., 2012). For example, in Reed et al. (2012), for some subtypes of work problems (e.g., distance = rate1 × time1 + rate2 × time2), comparing problems with arithmetic solutions resulted in an improved ability to solve similar problems, whereas comparing problems with algebraic solutions did not. In addition, comparing arithmetic problems did not lead to a learning transfer to algebraic problems or vice versa. Comparing problems of these two kinds did not lead to improvement on learning, either. These findings imply that when problems are structured differently, even though the elements involved and the solution methods are the same, their difficulty or complexity is different.

As the studies on analogical learning have demonstrated, learning through mapping or comparing example problems can be improved through deliberate guidance that highlights conceptual structures. In addition, coupling example problems of different complexities may facilitate structural and conceptual learning. However, the beneficial effects of such coupling are not well understood because they are not consistently identified.

## 4. The present study

Because the literature on how learners perform principle–example mapping is lacking, this study conducted an investigation of learners’ behavior under the condition of no formal instruction being provided. Three research goals were established. The primary goal was to examine the characteristics of principle–example mapping, hereinafter referred to as *PE mapping*. To enable a comparison, example–example mapping, hereinafter referred to as *EE mapping*, was also examined. Although principles are deemed important for relational learning (e.g., Goldstone and Son, 2005), empirical evidence supporting the benefits of PE mapping in this context is lacking. Therefore, the study initially investigated learners’ tendencies regarding structural mapping during learning tasks to accomplish this goal. Additionally, understanding the relative difficulty of PE and EE mapping can be valuable for educators interested in adopting these mapping approaches for instructional material planning. Thus, the second issue addressed in this study was the systematic investigation of the difficulty associated with PE and EE mapping.

The secondary goal aimed to explore the factors that may affect PE and EE mapping. Content complexity might play a role in relational learning, however, little research has delved into this aspect. Therefore, this study explored two sources of complexity, namely principles and examples, to determine whether and how they affect learning through mapping. *Complexity* was defined as the number of entities and relations within a form of content, focusing on structural learning (e.g., Halford, 1992). Simple principles had fewer elements and thus lower structural complexity, whereas complex principles had the opposite characteristics. Similarly, simple examples provided all required components for direct application of a principle, whereas complex examples included some given components but required finding others on the basis of available information regarding entities and relationships.

To examine the conception of conceptual structures, the participants’ responses to the unconstrained, free-mapping task were analyzed in terms of nonrelational and relational responses according to the structure-mapping theory (Gentner, 1983). *Nonrelational* responses were mappings that did not involve relational elements, such as entities, properties, or property relationships (e.g., a continuous space). *Relational* responses were mappings that involved relational elements, such as first-order (e.g., events occurring in a space) or higher-order relationships (e.g., events occurring in a space independent of events in another space). The connections between principle and example complexity and relational learning are discussed in greater detail in the “Predictions” section.

To summarize, the research questions for the above goals were as follows:

1. How do the connections established in PE mapping and EE mapping differ?
2. Is PE mapping more difficult than EE mapping?
3. Does the complexity of an example affect the results for Questions 1 and 2?
4. Does the complexity of a principle affect the results for Questions 1 and 2?

For instructors and learners to feel motivated to adopt PE mapping as a strategy, the effects of such mapping must be understood. Therefore, the final goal of the study was to explore the effects of PE mapping on learning. As previously indicated, the PE mapping approach was expected to facilitate structural learning; thus, the learning effect to be evaluated was concerned with the learners’ structural knowledge.

Researchers, however, do not have a consensus on how the outcomes of structural learning should be measured. Some have measured whether a learner could articulate connections among the entities involved in word problems. That is, they have measured the results or schemas of problem-solving (e.g., Bassok, 1990; Novick and Holyoak, 1991). While the acquisition of abstracted knowledge necessitates understanding individual entities and their interrelations, it also entails comprehending the meaning, properties, and constraints associated with these elements. In this study, these forms of knowledge were referred to as *structural* and *conceptual knowledge*, respectively. Research has revealed that acquiring one form of knowledge may not guarantee acquisition of the other (e.g., Rittle-Johnson and Alibali, 1999). Therefore, in assessing the effects of PE mapping in structural learning, both structural and conceptual knowledge should be considered.

For the final goal, the research questions investigated were as follows:

1. Is the conceptual knowledge acquired through PE mapping different from that acquired through EE mapping?
2. Is the structural knowledge acquired through PE mapping different from that acquired through EE mapping?
3. Does the complexity of an example modulate the differences of Questions 5 and 6?
4. Does the complexity of a principle modulate the differences of Questions 5 and 6?

### 4.1. Design

A mixed-design experiment was employed for the study. The participants were tasked with studying foundational materials related to the target content. They then completed a free-mapping and a guided-mapping task based on the assigned condition, followed by tests assessing their conceptual and structural knowledge.

The independent variables included mapping type (PE or EE), example complexity (simple or complex), and principle complexity (simple or complex). Mapping type and example complexity served as between-subjects variables and were combined to create five experimental conditions: principle–simple example (PEs), principle–complex example (PEc), simple example–simple example (EsEs), simple example–complex example (EsEc), and complex example–complex example (EcEc). Principle complexity was a within-subject variable; participants assigned to each experimental group completed tasks at each level of principle complexity.

Based on the study’s definition of complexity, Geometric and Poisson distributions were, respectively, chosen as the simple and complex principles. Examples were designed so that the simple examples required one step to obtain a solution and the complex examples required three steps. Demonstrations of the principles and worked examples at two complexity levels can be found in Appendices A2, A3.

The dependent variables were scores derived from the tasks the participants completed. For each principle, the following scores were assigned for every participant: mapping difficulty, nonrelational responses, relational responses, and the sum score of nonrelational and relational responses, conceptual knowledge, and structural knowledge. Further details are provided in the “Methods” section.

### 4.2. Predictions

The present study hypothesized that, in general, PE mapping would facilitate relational comparison whereas EE mapping would facilitate nonrelational comparison. Researchers have agreed that teaching principles offers the advantage of presenting the elements of a conceptual structure without irrelevant, distracting features (Goldstone and Son, 2005; Sloutsky et al., 2005; Koedinger et al., 2008). Although the abstractness of principles causes PE mapping to be more difficult than EE mapping, it can also effectively highlight the roles of the entities and relations among the entities involved in an example (Goldstone and Son, 2005). Consequently, PE mapping is more likely than EE mapping to lead to relational comparison and is less likely to lead to nonrelational comparison. These differences between PE and EE mapping, however, may not be substantial if a principle is simple.

Differing levels of example complexity may also affect mapping patterns. As example complexity increases, the likelihood of a learner employing relational comparison might also increase because the examples demonstrate the process of transformation to highlight the relationships among the entities, which can facilitate relational processing. When the complexity is higher in a PE pair, PE mapping is likely to yield even more relational comparisons and fewer nonrelational comparisons than EE mapping is. When the complexity is higher in an EE pair, the differences between the results of PE and EE mapping are likely less important because more relational comparisons are likely to be made in EE mapping.

This study also hypothesized that PE mapping would generally be more difficult than EE mapping. According to the structure-mapping theory (Gentner, 1983), regardless of whether they are applying PE or EE mapping, learners attempt to identify one element of a target that corresponds to a single element of the second target. When a learner knows nothing about a domain, they may consider any element of the first target to correspond to more than one element of the second. In EE mapping, a learner may more easily identify matches (e.g., Novick, 1988; Bassok and Olseth, 1995; Ross and Kilbane, 1997) when they consider the role of an element, that is, the predicate of an argument (Gentner, 1983), because the attributes or role of an element within a specific context can be more easily extracted. In PE mapping, however, a lack of context cues and the generality of the terms used to describe the principle can increase the difficulty of determining the attributes or roles of elements, which increases the difficulty of matching elements. Therefore, when the complexity of a principle is higher, the differences in difficulty between PE and EE mapping may become more significant.

The difficulty of PE and EE mapping may also change with the complexity of an example. A pair of items containing at least one complex example for PE or EE mapping is likely to be more difficult to map than a pair containing two simple and similar items. To comprehend and map such pairs, the learner must make additional inferences and transformations to render the two items comparable. Although undertaking such a process can be an opportunity for learning, it may also be perceived to be an additional challenge to the learner, particularly during the initial learning period. When the example complexity is higher in a PE pair, the difference in the difficulty of PE and EE mapping may become more significant (because PE mapping is generally more difficult than EE mapping). However, when example complexity is higher in an EE pair, the difference may become less significant (because the difficulty of EE mapping is higher).

The effects of mapping on structural and conceptual learning can be viewed as the cumulative outcome of two mapping tasks evaluated under the condition of no intervention. The aforementioned discussions indicate that as the principle complexity or example complexity within a PE pair increases, the benefits of relational processing due to the principle may also increase in PE mapping. However, at the same time, the disadvantage arising from the abstract nature of the principle for PE mapping may also intensify. It remains unclear how the positive and negative effects of principles interact with each other to influence the overall learning outcomes. As a result, no specific predictions were made regarding the differences between PE and EE mapping.

## 5. Methods

### 5.1. Participants

Undergraduate students were recruited through advertisements posted around the campuses of a small university. Students were considered eligible if they had not taken classes on the selected topics. The sample size was determined to detect a standardized effect size of at least 0.35, resulting in a target size 145. Details regarding this estimation are provided in the Supplementary materials. In the end, 149 participants took part in the study, but 11 participants were excluded from the analysis due to a high number of missing responses or failure to correctly follow the task instructions. The study included 103 female and 35 male participants; the numbers of female and male participants were similar across the five conditions.

### 5.2. Materials

Three sets of printed materials were used in this study. The contents were adapted from standard textbooks that were suitable for initial learning of the topics. The *prerequisite-study booklet* contained relevant probability concepts (e.g., random experiments, random variables, probability function and distribution, and expected values) that the participants could familiarize themselves with. The contents of the booklet are provided in Appendix A1.

The *work booklet* contained tasks used to measure the participants’ behavior of mapping. A version of the booklet was developed for each of the five experimental conditions. Each version comprised six pages: half of the pages presented tasks for the simple principle, and half presented tasks for the complex principle. The order of the tasks illustrating the two types of principles was counterbalanced within each condition. For each half of a booklet, the first page presented a principle and worked example or two worked examples. The PE and EE pairs were prepared for the simple and complex principles using the following criteria: One example of the PE and EE pairs was presented in the same context, and the second example of the EE pair was presented in a different context. Representative PE pairs are presented in Appendices A2, A3.

The second page of each half of a booklet contained a *free-mapping* task, and the remaining pages contained a *guided-mapping* task (examples provided in Appendices A4, A5). The free-mapping task was used to measure the participants’ behavior when minimal guidance was provided. The participants’ performances would indicate their natural tendencies or propensities with respect to mapping. In the task, the participants were asked to identify the commonalities and the differences between two targets (e.g., Markman and Gentner, 1996).

The guided-mapping task was used to measure the participants’ behavior when they received guidance. The participants were more likely to demonstrate their abilities of mapping when provided with guidance (e.g., Catrambone and Holyoak, 1990). Thus, this task was used to determine the difficulty of mapping. A list of 12 minor components of the target principle or example was provided for each principle. For each set of responses, the participants were asked to construct a stem for mapping by using the provided components and to identify a corresponding part in another target example. The numbers of components required to construct the stems were two, three, four, or five or more. For each size, up to three sets of responses could be given. This task was presented over two pages.

A third booklet contained two tasks for measuring the effect of mapping. This *test booklet* was the same for all experimental conditions. However, within each condition, the task order was counterbalanced with the mapping order. One of the tasks was the *multiple-choice task*, which was used to assess the participants’ conceptual understanding of the aspects of the target principles. The task comprised two parts, each of which contained five questions on one of the target principles. The order in which the two parts were presented was the same as that in which the target principles were presented in the work booklet. The questions were described in general terms, and only one option among four was correct.

The other task was the *word-problem task*, which was used to assess the participants’ knowledge of the overall conceptual structures of the two target principles. The task comprised two problems related to each of the two principles and two additional problems regarding other forms of distribution that were used as foils. One of the two problems regarding each of the two principles was a simple problem, and the other was a complex problem. These six word problems were presented in the same semirandom order for each experimental condition. The instructions specified that some of the problems could be solved by using one of the two target principles and that if a problem could be solved using one of these principles, only the equation associated with the principle needed to be provided.

### 5.3. Procedure

The study commenced after approval was granted by a research ethics committee. Groups of eight participants or fewer completed the experiment in a laboratory on a campus or a designated room in the library of another. The participants were seated with sufficient space between them to work independently and receive individual help from the experimenter if needed. The participants were randomly assigned to an experimental condition upon their arrival through the block randomization procedure and were not informed of which condition they had been assigned to. Both the experimenters and the participants were blinded to the research questions and hypotheses. The experimenters asked the participants to sign a consent form and comply with the requirements of the experiment. Afterwards, the participants studied the prerequisite-study booklet, completed the work booklet for their assigned condition, and then completed the test booklet within a single session. The booklets were provided all at once but the participants were instructed to work on one booklet at a time. The participants were encouraged to attempt to comprehend and memorize the learning materials but were allowed to view the pages containing the PE or EE pair as they completed the mapping tasks in the work booklet. Before the participants started working on the test booklet, the other two booklets were retrieved. No feedback was provided to the participants on any of the tasks. The experimenters emphasized to the participants that they should complete the tasks in order and attempt to answer every question even if the questions were difficult. For each principle, the time allocated for the prerequisite learning, target learning, free-mapping task, and guided-mapping task was 5, 5, 10, and 15 min, respectively. This allocation was to ensure that the participants would spend a sufficient amount of time on each part of the experiment. In addition, 20 min were allocated to complete the test booklet. At the end of the experiment, each participant received a monetary reward of $5 for their participation.

### 5.4. Analysis

The PROC MIXED of SAS was the primary procedure used for the analyses. Because the order variable was not a variable of interest for this study and did not affect the results, it was excluded from the analyses. The model for the free-mapping task included the following variables as fixed effects: principle, mapping, and relation, with principle and relation used as within-subject variables. For the other tasks, the relation variable was not included. The Kenward–Roger method was used to correct the estimates (Littell et al., 2006), and an unstructured option was chosen for the covariance structure. Separate tests were conducted for each principle based on the models. To test the effect of mapping type, the two PE conditions were compared with the three EE conditions. To determine the effect of example complexity, three tests were conducted. In the first, the two PE conditions were compared because the EE conditions were held constant (i.e., the complexity was higher in the PE pair). In the other two tests, the EsEs versus EsEc and the EsEs versus EcEc conditions were compared because the PE conditions were held constant (i.e., the complexity was higher in the EE pair). Type I error rates were controlled at 0.05 for each principle at the family level, and therefore, the alpha values were also 0.05 for the variable mapping type and example complexity. When analyses were conducted on the subcategories of responses (e.g., relational and nonrelational responses), the error rates were divided by the number of categories. Finally, the effect of principle complexity was analyzed by using the overall patterns of the results of the analyses.

To calculate effect sizes, two approaches were used. For the free-mapping task, because the numbers of responses provided by the participants were different (i.e., no fixed scale), the effect sizes and their confidence intervals were estimated using the *bootES* package developed by Kirby and Gerlanc (2013). The bias-corrected-and-accelerated bootstrap method was used to estimate Hedges’s *g* and its 95% confidence interval on the basis of 2,000 resamples. The estimates obtainable through these methods are similar to Cohen’s *d* but have a higher accuracy (Kelley, 2005). For the guided-mapping task and the two tests, raw estimated differences (presented as raw *d*) between or among groups and their 95% confidence intervals were reported. These estimations were based on the analytical models and accounted for covariance among the variables. If a confidence interval contained zero, it is not reported for the sake of space.

## 6. Results and discussion

### 6.1. Free-mapping task

The responses to the questions were segmented and coded based on the ideas that were expressed regarding each principle. The score for each principle was calculated as the number of coded responses for the participants. A random sample of 25% of the participants was drawn (*n* = 35), and the responses of the sample were independently coded by two coders. For the simple principle, 161 pairs of codes were identified and simple Kappa = 0.87, ASE = 0.04, two-tailed *p* < 0.001. For the complex principle, 154 pairs of codes were identified and simple Kappa = 0.76, ASE = 0.04, two-tailed *p* < 0.001. For all data, differences in the codes assigned by the two coders were resolved through discussion or the decision of the researcher.

The coded responses were classified into the following categories: entity, property, property relation, first-order relation, higher-order relation, and other. The final category was a combination of several codes, namely no similarities or differences; do not know; blank; and responses that were uninformative, incomprehensible, or conceptually irrelevant. To analyze the mapping tendency, the first three categories were combined to form a nonrelational category, and the first-order and higher-order relation categories were combined to form a relational category. For each principle, two scores were calculated as the number of summated codes for these new categories.

The data as presented in Table 1 revealed that the category sums were similar between the two principles and the PE conditions generally had lower score sums than the EE conditions did on both principles. Additionally, more relational than nonrelational responses were provided under the PE conditions, whereas the opposite pattern was shown under the EE conditions, particularly when the complex principle was presented. For the simple principle, the planned test of the nonrelational responses revealed a mapping effect in which fewer responses were provided under the PE conditions than under the EE conditions, *t*(133) = −3.34, *SE* = 0.22, two-tailed *p* = 0.001, Hedges’s *g* = −0.58, CI [−0.85, −0.21]. The test of the relational responses did not reveal a mapping effect, *t*(133) = 0.65, *SE* = 0.21, two-tailed *p* > 0.10, Hedges’s *g* = 0.11, CI [−0.21, 0.47]. Furthermore, the tests of the example effect showed no significant differences (two-tailed *p*s > 0.10).

**Table 1:** Mean number and standard deviation of responses by principle and experimental condition on the free-mapping task.

		Nonrelational		Relational		Sum		Total	
Mapping	*n*	*M*	*SD*	*M*	*SD*	*M*	*SD*	*M*	*SD*
Simple									
PEs	27	1.41	1.01	2.07	0.83	3.48	1.42	3.96	1.26
PEc	28	1.14	1.08	2.00	1.36	3.14	1.58	3.61	1.29
EsEs	28	2.14	1.72	2.14	1.38	4.29	1.90	5.43	1.97
EsEc	28	1.89	1.10	1.96	1.40	3.86	1.60	4.50	1.64
EcEc	27	1.96	1.19	1.59	0.97	3.56	1.37	4.48	1.53
Average	138	1.71	1.29	1.96	1.21	3.67	1.61	4.40	1.66
Complex									
PEs	27	1.19	1.55	2.11	1.28	3.30	1.32	3.52	1.37
PEc	28	1.39	1.50	1.82	1.49	3.21	1.32	3.50	1.32
EsEs	28	3.18	2.04	1.11	0.88	4.29	2.03	5.04	2.12
EsEc	28	2.64	1.50	1.29	1.15	3.93	1.18	4.57	1.35
EcEc	27	2.26	1.16	1.63	1.15	3.89	1.37	4.11	1.25
Average	138	2.14	1.73	1.59	1.24	3.72	1.51	4.15	1.61

PEs = principle–simple example; PEc = principle–complex example; EsEs = simple example–simple example; EsEc = simple example–complex example; EcEc = complex example–complex example. Sum: combination of nonrelational and relational responses. Total: including “others” responses.

For the complex principle, the planned test of the mapping effect indicated that fewer nonrelational responses were provided under the PE conditions than under the EE conditions, *t*(133) = −5.12, *SE* = 0.27, two-tailed *p* < 0.001, Hedges’s *g* = −0.89, CI [−1.25, −0.51], and more relational responses were provided under the PE conditions than under the EE conditions, *t*(133) = 2.98, *SE* = 0.21, two-tailed *p* < 0.01, Hedges’s *g* = 0.52, CI [0.16, 0.89]. The other tests of the example effect did not produce any significant results (two-tailed *p*s > 0.10).

To summarize, the mapping propensities elicited by PE mapping and EE mapping differed. The learners’ mapping behaviors were affected by the interaction of mapping type and principle complexity (Question 4). When a simple principle was being learned, PE mapping led to fewer nonrelational comparisons than EE mapping but the same number of relational comparisons. When a complex principle was being learned, PE mapping led to fewer nonrelational comparisons than EE mapping but more relational comparisons (Question 1). These findings verify the predictions regarding mapping type and principle complexity.

The results regarding example complexity showed that for both types of principles, variations in the example complexity had little or no influence on the participants’ mapping tendencies (Question 3). These findings support the prediction regarding simple principles but not that regarding complex principles. This finding of a nonsignificant effect of example complexity on relational mapping during the free-mapping task might be explained by the multifarious behaviors of the participants. The participants may have avoided mapping content they were not confident about; that is, the participants assigned to the five conditions may have mapped content that did not require transformation and thus had similar performances. In addition, when the participants mapped transformational content, some merely indicated the conceptual categories the content belonged to. For example, a calculation was required to determine the frequency of an event in one worked example; however, some participants simply expressed that the two examples being compared mentioned “event frequency” or had different event frequencies. These responses were coded as nonrelational. Therefore, the effect of example complexity might have been partly diminished by how the participants articulated the mappings.

Learners’ mapping behaviors are likely affected most by mapping type and principle complexity. Mapping two examples tends to lead to more nonrelational comparisons regardless of whether the underlying principle of the examples is simple or complex. Mapping a principle onto an example of the principle tends to lead to more relational comparisons when the principle is more complex. When the principle is simple, the effects of PE and EE mapping on relational comparison are similar.

### 6.2. Guided-mapping task

The scores for the guided-mapping task were determined for each principle as follows. Each pair of responses constructed for PE mapping or EE mapping was determined to have no correct, a partially correct, or a completely correct correspondence. If a pair had a partially correct or completely correct correspondence, the correspondence was considered correct. Notably, the scoring was focused on the correct rather than incorrect mapping the participants completed. If a correct correspondence was similar to an example provided in the instructions, that response was not counted. In addition, if duplicate correct correspondences were identified because partially correct responses had been provided, only one correspondence was counted. For each principle, the maximum possible score per participant was 12. The alpha coefficient, calculated using the R package *psych* (Revelle, 2019), on the tetrachoric correlations among the 12 items was 0.80 for the simple principle and 0.83 for the complex principle.

As the mean total scores in Table 2 demonstrate, the two types of principles had similar mean scores, and the PE conditions on average had similar mean scores to the EE conditions for both principles. However, the PEc and EsEc conditions had slightly lower mean scores than the other conditions did for both principles. The analyses based on the total scores yielded the following results. Regarding the simple principle, the planned test of the mapping effect indicated similarity in performance for PE and EE mapping, *t*(131) = −0.64, *SE* = 0.30, two-tailed *p* > 0.10, raw *d* = −0.19, CI [−0.78, 0.40]. The analyses of the example effect indicated that only the EsEs condition led to a better performance than the EsEc condition did, *t*(131) = 2.76, *SE* = 0.47, two-tailed *p* < 0.01, raw *d* = 1.29, CI [0.37, 2.21].

**Table 2:** Mean number and standard deviation of correct elements by principle and experimental condition on the guided-mapping task.

		Corresponding		Noncorresponding		Total	
Mapping	*n*	*M*	*SD*	*M*	*SD*	*M*	*SD*
Simple							
PEs	27	2.96	1.97	1.37	1.24	4.33	1.90
PEc	28	2.29	1.56	1.04	1.04	3.32	1.59
EsEs	28	3.50	1.75	0.89	1.10	4.39	1.87
EsEc	28	2.62	1.30	0.46	0.65	3.08^b^	1.55
EcEc	27	3.85	1.75	0.70	0.91	4.56	1.60
Average	138	3.04	1.75	0.90	1.04	3.94	1.79
Complex							
PEs	27	3.33	1.82	0.44	0.85	3.78	2.04
PEc	28	2.56	1.67	1.07	1.30	3.63^a^	2.17
EsEs	28	4.00	1.44	0.79	1.13	4.79	1.52
EsEc	28	2.44	1.12	0.33	0.55	2.78^a^	1.25
EcEc	27	3.96	1.43	0.56	0.97	4.52	1.76
Average	138	3.26	1.63	0.64	1.02	3.90	1.89

PEs = principle–simple example; PEc = principle–complex example; EsEs = simple example–simple example; EsEc = simple example–complex example; EcEc = complex example–complex example.

a One participant with missing data was excluded.

b Two participants with missing data were excluded.

With respect to the complex principle, the planned test did not reveal a mapping effect, *t*(131) = −1.04, *SE* = 0.31, two-tailed *p* > 0.10, raw *d* = −0.33, CI [−0.94, 0.29]. However, the tests revealed an example effect for the EsEs versus the EsEc condition, *t*(131) = 4.19, *SE* = 0.48, two-tailed *p* < 0.001, raw *d* = 2.01, CI [1.06, 2.96].

Even though no mapping effect was discovered from the overall scores, the participants in the PE conditions likely had an advantage over those in the EE conditions for the noncorresponding content because the latter did not have direct access to it. For the complex principle, for example (see Appendix A3), the first condition presented in the PE pairs was not mentioned in the EE pairs. To investigate the possible advantage, the total score was divided into a score for corresponding content and a score for noncorresponding content. The score for the noncorresponding content was calculated as follows. Of the 16 components provided for each principle in the task, three for the simple principle and five for the complex principle did not have correspondences in the examples. If a correct response contained one or more of these components, it was counted as a case of noncorresponding mapping; other responses were counted as cases of corresponding mapping.

As presented in Table 2, the responses were predominantly corresponding responses. The participants in the PE conditions tended to have lower performance than those in the EE conditions did on the corresponding content for the simple principle, *t*(131) = −2.40, *SE* = 0.29, two-tailed *p* = 0.02 raw *d* = −0.71, CI [−1.29, −0.12], and for the complex principle, *t*(131) = −1.97, *SE* = 0.27, two-tailed *p* = 0.051 raw *d* = −0.52, CI [−1.05, 0.001]. However, on the noncorresponding content, the participants in the PE conditions had higher performance than the participants in the EE conditions did for the simple principle, *t*(132) = 2.94, *SE* = 0.18, two-tailed *p* < 0.01, raw *d* = 0.52, CI [0.17, 0.87]. These findings indicated that PE mapping had an advantage over EE mapping regarding noncorresponding content even though the test was not significant for the complex principle. This advantage compensated for the lower performance on the corresponding content and thus the overall performance on the task was similar between PE and EE mapping.

The results for the overall task show that the difficulty of PE mapping and EE mapping did not differ (Question 2). Furthermore, this similarity was not affected by principle complexity (Questions 4). Additional analysis, however, revealed a differential effect in the difficulty of PE and EE mapping in relation to corresponding and noncorresponding content. The performances on the two kinds of content also uncovered a principle effect. The finding that, on average, the PE conditions had higher mean than the EE conditions on the noncorresponding content with the simple principle supported the conjecture that the principle in PE mapping serves as a model for learning. This modeling effect, nevertheless, was somehow weakened as the principle complexity increased. Because the content variable was not manipulated in this study, it is difficult to determine whether this observation was due to the increased abstractness effect of the principle. Further evidence regarding the modeling effect is presented in the subsequent discussion on the example effect.

The finding that the PE conditions had a lower mean than the EE conditions on the corresponding content may be explained by (1) PE mapping leading to more noncorresponding comparisons, (2) PE mapping being more difficult than EE mapping for this kind of content, or (3) both. Nevertheless, the results for the complexity principle, in which no mapping effect was found in the overall task and the noncorresponding content, suggested that PE mapping was more difficult than EE mapping in relation to the corresponding content. This evidence supports the prediction on a mapping effect under certain conditions. To gain a complete understanding of how the content variable influences the mapping effect, future research should systematically manipulate the variable.

With respect to example complexity, for the overall task, EsEs mapping was easier than EsEc mapping was with both principles. In other words, PE mapping was less difficult than EE mapping when an EsEc pair was used in the latter (Question 3). These findings partially support the prediction that when a pair contain items of different complexities (i.e., PEc or EsEc), the difficulty of mapping would be higher. Notably, the effect of example complexity (PEs vs. PEc) was weak when the learners were required to employ PE mapping. This weaker effect may have occurred because of the modeling effect due to the presence of the principle. Although the example effect when EE mapping was used is in line with that reported in the literature (e.g., Reed and Bolstad, 1991), the effect when PE mapping was used requires further investigation to be characterized.

To recapitulate, PE mapping may not be more difficult than EE mapping. Factors such as content characteristics, example complexity, and principle complexity may influence the difficulty of mapping. Although PE mapping of corresponding elements is more difficult than EE mapping, such mapping may be as easy as or easier than EE mapping for noncorresponding elements. Example complexity may increase or decrease the difference in difficulty between these forms of mapping, depending on whether complexity is varied within the PE or EE pair. Finally, for more complex principles, the difference in the difficulty between these two forms of mapping may be smaller.

### 6.3. Multiple-choice task

For each principle, the participant score was calculated as the number of correctly answered questions. The mean scores are presented in Table 3. The tetrachoric correlations between the test items for each principle were used to calculate reliability. The resultant alpha coefficient was nearly zero for both principles. Analysis of variance components in which the participants and items were considered random factors and the principle was considered a fixed factor was performed. The results revealed that the Participant × Item interaction accounted for a large proportion of the variance in the data compared with the portion accounted for by the participant and item effects (0.215 vs. 0.003 and 0.033). This indicates that some participants had unconventional response patterns for the task that were not associated with the item difficulty. Rather, they indicate that some participants had learned some aspects of the principle more effectively than other aspects. Therefore, the correlation between any two items was low, which resulted in a low alpha coefficient.

**Table 3:** Mean number and standard deviation of correct responses by principle and experimental condition on the multiple-choice task.

		Simple		Complex	
Mapping	*n*	*M*	*SD*	*M*	*SD*
PEs	27	1.96	0.98	3.11	1.34
PEc	28	1.86	1.15	2.56^a^	1.05
EsEs	28	1.46	0.88	2.89	0.74
EsEc	28	1.65^b^	1.06	2.70^a^	0.99
EcEc	27	1.89	1.05	2.48	0.98
Average	138	1.76	1.03	2.75	1.05

PEs = principle–simple example; PEc = principle–complex example; EsEs = simple example–simple example; EsEc = simple example–complex example; EcEc = complex example–complex example.

a One participant with missing data was excluded.

b Two participants with missing data were excluded.

To determine the effect of these unconventional responses, another analysis was conducted for the “reliable” participants. Information regarding how these participants were identified is provided in the Supplementary materials. The alpha coefficient for the participants with normal response patterns was 0.73 (*n* = 78) for the simple principle and 0.65 (*n* = 87) for the complex principle. The results indicate that the normal response patterns could play a key role in determining the reliability of task performance.

Although the unconventional participants led to the measures having lower reliability, their learning progresses were not invalid. For this reason, an analysis that included the scores of all participants was conducted. The data as presented in Table 3 indicated that the simple principle had a lower mean score than the complex principle did. This was likely caused by the participants misunderstanding the concepts of experiment and trial, with the detrimental effect of this misunderstanding being larger for the simple principle. The effects of mapping type and example complexity were negligible. Because the participants assigned to the five conditions had differing performance on the guided-mapping task, a proportion-correct score for the overall task was used as a covariate in the analysis. The planned test of the mapping effect uncovered similar performance for the participants in the PE and EE conditions for the simple principle, *t*(131) = 1.37, *SE* = 0.18, two-tailed *p* > 0.10, raw *d* = 0.25, CI [−0.11, 0.60], as well as for the complex principle, *t*(131) = 0.82, *SE* = 0.18, two-tailed *p* > 0.10, raw *d* = 0.15, CI [−0.21, 0.51]. No example effect was identified for either principle (two-tailed *p*s > 0.05).

The same set of analyses was next performed for the conventional samples to enable a comparison. See Table 4 for the means of these samples. Because some removed cases differed with the principles, separate analyses were performed for each principle. The results were similar to those obtained in the previous set of analyses; therefore, they are not presented here. Overall, the two sets of analyses demonstrated that although the participants handled the content differently, as indicated by their test item scores, their overall learning outcomes were not affected differently by the factors investigated in this study.

**Table 4:** Mean number and standard deviation of correct responses with odd-response patterns removed by principle and experimental condition on the multiple-choice task.

	Simple			Complex		
Mapping	*n*	*M*	*SD*	*n*	*M*	*SD*
PEs	18	2.17	1.10	19	3.11	1.49
PEc	21	1.86	1.24	19	2.63	1.01
EsEs	11	1.64	1.21	17	2.65	0.93
EsEc	14	1.43	1.16	16	2.44	1.03
EcEc	14	1.93	1.21	16	3.00	0.73
Average	78	1.83	1.18	87	2.77	1.09

PEs = principle–simple example; PEc = principle–complex example; EsEs = simple example–simple example; EsEc = simple example–complex example; EcEc = complex example–complex example.

These analyses reveal that PE mapping and EE mapping resulted in similar increases in conceptual knowledge (Question 5). This similarity was not disrupted by example complexity (Question 7) or principle complexity (Question 8). A further discussion of these findings is presented with that on structural knowledge in the next section.

### 6.4. Word-problem task

An overall task score and two principle scores were calculated for each participant based on the number of problems they correctly solved. Because the problems of the task involved three sets of principles and only two items were included for each set, an alpha coefficient was calculated for the overall task with the tetrachoric correlations. The reliability coefficient was 0.80 for the overall task.

As shown in Table 5, the overall mean scores were low, and they were close to the sum scores of the two principles, which indicates the participants rarely solved the filler problems. The data also reveal that the simple principle had a higher mean score than the complex principle did, and an example effect may have been present. For the analysis, the two principle scores and the covariate proportion-correct scores from the guided-mapping task were included. The planned tests did not reveal any effects of mapping type or example complexity (two-tailed *p*s > 0.10), with the exception of the PEs condition leading to a better performance than the PEc condition did for the complex principle, *t*(131) = 2.95, SE = 0.16, two-tailed *p* < 0.01, raw *d* = 0.47, CI [0.16, 0.79].

**Table 5:** Mean number and standard deviation of correct responses by principle and experimental condition on the word-problem task.

		Simple		Complex		Whole task	
Mapping	*n*	*M*	*SD*	*M*	*SD*	*M*	*SD*
PEs	27	1.07	0.96	0.81	0.56	1.93	1.24
PEc	28	0.93	0.86	0.33^a^	0.55	1.26^a^	1.02
EsEs	28	1.29	0.81	0.68	0.55	2.07	1.30
EsEc	28	1.04^b^	0.92	0.41^a^	0.64	1.44^c^	1.36
EcEc	27	1.19	0.88	0.48	0.70	1.67	1.33
Average	138	1.10	0.88	0.54	0.62	1.68	1.27

PEs = principle–simple example; PEc = principle–complex example; EsEs = simple example–simple example; EsEc = simple example–complex example; EcEc = complex example–complex example.

a One participant with missing data was excluded.

b Two participants with missing data were excluded.

c Three participants with missing data were excluded.

Overall, the results indicate that the amount of structural knowledge the participants acquired through PE and EE mapping was similar (Question 6). This similarity, however, was affected by the interaction between example complexity (Question 7) principle complexity (Question 8). For learning the simple principle, example complexity did not affect the similarity. For learning the complex principle, PE mapping led to less gain than EE mapping when example complexity was increased in the PE pair.

The impact of mapping on learning could be attributed to the cumulative effects of the two mapping tasks. Since guided mapping was employed as the second task completed by the participants, it is likely to have influenced the test results. As demonstrated by the findings, participants in both the PE and EE conditions exhibited comparable performance on the guided-mapping task, regardless of the type of principles involved. This suggests that both forms of mapping resulted in similar learning outcomes when the assigned mapping tasks were completed. Consequently, this observation may elucidate why there were analogous gains in conceptual and structural knowledge for both forms of mapping, irrespective of the principle types.

An effect of example complexity was observed for both types of principles when the participants in the EE mapping conditions completed the guided-mapping task. However, this effect was not observed for the two tests used to measure conceptual and structural knowledge. On the other hand, an example effect on structural knowledge gains was identified when PE mapping was used to learn the complex principle. These results showed that example complexity and principle complexity had limited effect on the learning of conceptual and structural knowledge. The reason might be the following. During the guided-mapping task, the participants were asked to construct the stems for mapping and identify the corresponding components from the target example. Such a task is likely more challenging than the free-mapping task. However, because the guided-mapping task did not include formal instruction, the learning gains from the task were limited. The participants’ low to moderate performance on the two tests provides some evidence to support this speculation. Little example effect was discovered through the tests, which was likely because the participants assigned to the different conditions had not yet learned the content well enough that differential effects could be identified.

Although PE mapping and EE mapping may elicit different mapping behaviors with principles of varying complexity and differ in difficulty with content of different features, PE mapping can result in similar, if not higher, levels of structural and conceptual knowledge about the principle compared to EE mapping. In addition, these findings remain robust even when the principle complexity increases.

## 7. General discussion

The current study investigated the characteristics of connecting to-be-learned information (i.e., principles and examples) by mapping their entities, relationships, and conceptual structures. The findings contribute to the knowledge of analogical reasoning and learning by mapping one abstract principle onto one concrete example. Through this line of research, learning scientists can identify additional means of enabling learners to overcome the obstacles of comprehending abstract or general information and elucidate the interrelations among the individual concepts in formal structures. Although this line of research remains at an early stage, the current findings may provide some theoretical and instructional insight.

### 7.1. Principle–example mapping may be as effective as example-example mapping

The study findings seem to support that principle–example mapping is as effective as example–example mapping. In the guided-mapping task, the participants in the PE conditions compared fewer corresponding components but generally compared more noncorresponding components than did those in the EE conditions. Their higher number of comparisons of noncorresponding components compensated for the lower number of comparisons of corresponding components, which led the participants in the PE and EE conditions to have a similar overall performance. In addition, this similarity in their performance was not affected by principle complexity. These findings indicate that the two forms of mapping are generally equally effective for learning simple materials, such as those employed in this study. More importantly, the findings support the proposal that principle–example mapping can overcome the learning difficulty resulting from the abstractness of a principle.

Notably, in the guided-mapping task, the participants were able to freely determine what they should map. They are likely to have avoided mapping content that was difficult for them. If they had been directed to map all specified components, the findings may have been different. Therefore, future studies should use different guided-mapping tasks to test whether the conclusion regarding an equal effect is generalizable.

### 7.2. Principle–example mapping has a modeling effect

Several findings suggest that principles have a modeling effect in principle–example mapping. On the guided-mapping task, the participants in the EsEc condition had worse performance than those in the EsEs condition did. However, the participants in the PEc condition did not have worse performance than did those in the PEs condition. These findings were robust because they did not interact with principle complexity. Other findings revealed that the participants in the PE conditions had better performance than did those in the EE conditions with respect to mapping noncorresponding content. These findings imply that, when a learner attempts PE mapping, they can compare an example with the principle it illustrates and comprehend how the differences between the principle and example can be explained by the principle because the principle provides complete, nondistracting information.

With respect to how these findings can be applied in instruction, when a variety of worked examples or word problems are used in a lesson, they should be compared with the principle they illustrate. Merely mapping examples or problems of different levels of complexity may not result in the learner comprehensively understanding the underlying principle. This is particularly true when instruction time is limited or students are required to review examples or problems outside of class (e.g., Chi et al., 1989).

### 7.3. Principle–example mapping facilitates structural learning

Much of the evidence of this study support that principle–example mapping facilitates structural learning. In the free-mapping task, PE mapping of the complex principle led to the participants making more relational comparisons than EE mapping did, whereas EE mapping led to the participants making more nonrelational comparisons regardless the complexity of the principle. Although the relational advantage of PE mapping was not obtained with the simple principle, the participants in the hybrid mapping conditions were more likely to complete more relational than nonrelational comparisons, whereas those in the traditional mapping conditions were more likely to complete more nonrelational than relational comparisons, as indicated by the data presented in Table 1. These findings align with the argument that principled information facilitates relational or structural learning (e.g., Goldstone and Son, 2005).

Other evidence supporting the facilitating effect of principle–example mapping come from the results of the conceptual and structural tests. In the literature, abstract information has been reported to be difficult to learn (e.g., Reed and Bolstad, 1991). However, in the current study, the two forms of mapping yielded comparable learning outcomes in conceptual and structural knowledge. This suggests that when a learner employs PE mapping, the difficulties associated with processing abstract information are compensated for by the utility afforded by a principle for processing relational information. Although EE mapping does not have this disadvantage, the resulting learning outcomes are not superior to those of PE mapping. This implies that the level of relational learning achieved may not be as high as that in PE mapping.

### 7.4. Instructional implications

The learning gains resulting from the participants completing the mapping tasks were somewhat limited. Therefore, some instructional intervention is required for the hybrid mapping approach to be sufficiently effective. In particular, rich and specific instructional support for novice learners are especially important (e.g., Bokosmaty et al., 2015). Research has provided suggestions regarding how mapping tasks can be effectively utilized. Firstly, transitioning from example–example mapping to principle–example mapping can be beneficial for understanding the principle. Some theorists have proposed (Halford, 1992; Rattermann and Gentner, 1998) that initial learning most likely begins with understanding individual entities and subsequently their relations with other entities or the relations between sets of local conceptual structures. Learning a principle by comparing two concrete examples and then comparing the principle with a concrete example may mimic this process. This approach can address the potential difficulty of principle–example mapping under certain conditions by guiding the learner to identify specific examples to which the principle may be applied. In addition, comparing such examples may enable the learner to identify key entities and their roles and properties (Alfieri et al., 2013). Through example–example mapping, the relationships among key entities may be noted, and through additional principle–example mapping, the properties and roles of the entities and the relationships among them can be explicitly understood. Therefore, even if a learner does not or cannot identify the general principle underlying a set of examples (e.g., Mayer, 2004), they can still understand the principle through principle–example mapping.

Secondly, transitioning from free mapping to guided mapping can be beneficial for motivation for learning and learning for understanding. In the present study, when the participants completed a free-mapping task, their responses were occasionally general. When their responses regarding the transformational content were general, determining whether they understood the exact relations among the transformational elements became difficult. Even when constraints are imposed, as they were in the present study’s guided-mapping task, participants may avoid mapping difficult content, such that requiring inferences and transformations. Such avoidance could prevent learners from learning critical elements of a domain. Therefore, when designing instructional and learning tasks, the amount of guidance provided should be considered. For tasks aimed at enabling learners to explore the structure of a domain, a free-mapping task would be more likely to encourage such exploration (e.g., Lamnina and Chase, 2019). On the other hand, when the goal is to help learners understand the elements of a conceptual structure, a guided-mapping task with specific requirements may lead to more effective and efficient learning. In addition, instruction involving free mapping followed by guided mapping may encourage learners to engage in more active learning.

### 7.5. Limitations and future research

In this study, two factors regarding principles and examples were investigated, and therefore, the findings likely elucidate only some aspects of principle–example mapping. Future research can expand on the present study’s findings by, for example, including principles from nonscience domains or examples that do not involve problem-solving. In addition, this study analyzed the characteristics and effects of principle–example mapping when no instructional intervention was provided. An analysis of the effects of mapping when an intervention is implemented would deepen the understanding of the effectiveness of the hybrid approach.

Future research should promptly address several issues of significance. One such issue is how principle–example mapping can be adapted for instructional purposes and whether the effects of such implementations can be generalized to the target populations of learners. Another is how the effect of principle–example mapping implemented as an instructional strategy differs from those of other forms of instruction, such as providing principled explanations or intermixing instructional explanations with worked examples. Research should also consider other issues that are more theoretical. For example, researchers should answer the following questions: Is there a difference between the cognitive processes evoked in principle–example mapping and in example–example mapping? How do these cognitive processes differ, and what do they imply? These research questions, albeit incomprehensive, should be investigated to achieve a complete understanding of the hybrid approach proposed in this study.

## Data availability statement

The raw data supporting the conclusions of this article will be made available by the authors, without undue reservation.

## Ethics statement

The studies involving humans were approved by Research Ethics Committee National Taiwan University. The studies were conducted in accordance with the local legislation and institutional requirements. The participants provided their written informed consent to participate in this study.

## Author contributions

HL contributed to conception of the study, research design, data collection, and analysis, and manuscript writing.

## Funding

This research was supported in part by Tzu Chi University, Grant TCMRC-P-100012.

## Conflict of interest

The author declares that the research was conducted in the absence of any commercial or financial relationships that could be construed as a potential conflict of interest.

## Publisher’s note

All claims expressed in this article are solely those of the authors and do not necessarily represent those of their affiliated organizations, or those of the publisher, the editors and the reviewers. Any product that may be evaluated in this article, or claim that may be made by its manufacturer, is not guaranteed or endorsed by the publisher.

## References

[ref1] Alfieri L. Nokes-Malach T. J. Schunn C. D. (2013). Learning through case comparisons: a meta-analytic review. Educ. Psychol. 48, 87–113. doi: 10.1080/00461520.2013.775712

[ref2] Bassok M. (1990). Transfer of domain-specific problem-solving procedures. J. Exp. Psychol. Learn. Mem. Cogn. 16, 522–533. doi: 10.1037/0278-7393.16.3.522

[ref3] Bassok M. Olseth K. L. (1995). Object-based representations: transfer between cases of continuous and discrete models of change. J. Exp. Psychol. Learn. Mem. Cogn. 21, 1522–1538. doi: 10.1037/0278-7393.21.6.1522

[ref4] Belenky D. M. Schalk L. (2014). The effects of idealized and grounded materials on learning, transfer, and interest: an organizing framework for categorizing external knowledge representations. Educ. Psychol. Rev. 26, 27–50. doi: 10.1007/s10648-014-9251-9

[ref5] Bokosmaty S. Sweller J. Kalyuga S. (2015). Learning geometry problem solving by studying worked examples: effects of learner guidance and expertise. Am. Educ. Res. J. 52, 307–333. doi: 10.3102/0002831214549450

[ref6] Catrambone R. Holyoak K. J. (1990). Learning subgoals and methods for solving probability problems. Mem. Cogn. 18, 593–603. doi: 10.3758/BF03197102, PMID: 2266861

[ref7] Chi M. T. Bassok M. Lewis M. W. Reimann P. Glaser R. (1989). Self-explanations: how students study and use examples in learning to solve problems. Cogn. Sci. 13, 145–182. doi: 10.1207/s15516709cog1302_1

[ref8] Cummins D. D. (1992). Role of analogical reasoning in the induction of problem categories. J. Exp. Psychol. Learn. Mem. Cogn. 18, 1103–1124. doi: 10.1037/0278-7393.18.5.1103

[ref9] Day S. B. Motz B. A. Goldstone R. L. (2015). The cognitive costs of context: the effects of concreteness and immersiveness in instructional examples. Front. Psychol. 6:1876. doi: 10.3389/fpsyg.2015.01876, PMID: 26648905 PMC4665226

[ref10] Dixon J. A. (2005). “Mathematical problem solving: the roles of exemplar, schema, and relational representations” in Handbook of mathematical cognition. ed. Campbell J. I. D. (New York, NY US: Psychology Press), 379–395.

[ref11] Dumas D. Alexander P. A. Grossnickle E. M. (2013). Relational reasoning and its manifestations in the educational context: a systematic review of the literature. Educ. Psychol. Rev. 25, 391–427. doi: 10.1007/s10648-013-9224-4

[ref12] Fyfe E. R. McNeil N. M. Son J. Y. Goldstone R. L. (2014). Concreteness fading in mathematics and science instruction: a systematic review. Educ. Psychol. Rev. 26, 9–25. doi: 10.1007/s10648-014-9249-3

[ref13] Gentner D. (1983). Structure-mapping: a theoretical framework for analogy. Cogn. Sci. 7, 155–170. doi: 10.1207/s15516709cog0702_3

[ref14] Gentner D. (1989). “The mechanisms of analogical learning” in Similarity and analogical reasoning. eds. Vosniadou S. Ortony A. (New York, NY: Cambridge University Press), 199–241.

[ref15] Gentner D. Loewenstein J. Thompson L. (2003). Learning and transfer: a general role for analogical encoding. J. Educ. Psychol. 95, 393–408. doi: 10.1037/0022-0663.95.2.393

[ref16] Gerjets P. Scheiter K. Catrambone R. (2006). Can learning from molar and modular worked examples be enhanced by providing instructional explanations and prompting self-explanations? Learn. Instr. 16, 104–121. doi: 10.1016/j.learninstruc.2006.02.007

[ref17] Gick M. L. Holyoak K. J. (1980). Analogical problem solving. Cogn. Psychol. 12, 306–355. doi: 10.1016/0010-0285(80)90013-4

[ref18] Goldstone R. L. Son J. Y. (2005). The transfer of scientific principles using concrete and idealized simulations. J. Learn. Sci. 14, 69–110. doi: 10.1207/s15327809jls1401_4

[ref19] Goldwater M. B. Schalk L. (2016). Relational categories as a bridge between cognitive and educational research. Psychol. Bull. 142, 729–757. doi: 10.1037/bul0000043, PMID: 26950007

[ref20] Halford G. S. (1992). Analogical reasoning and conceptual complexity in cognitive development. Hum. Dev. 35, 193–217. doi: 10.1159/000277167

[ref21] Kelley K. (2005). The effects of nonnormal distributions on confidence intervals around the standardized mean difference: bootstrap and parametric confidence intervals. Educ. Psychol. Meas. 65, 51–69. doi: 10.1177/0013164404264850

[ref22] Kirby K. N. Gerlanc D. (2013). BootES: an R package for bootstrap confidence intervals on effect sizes. Behav. Res. Methods 45, 905–927. doi: 10.3758/s13428-013-0330-5, PMID: 23519455

[ref23] Koedinger K. R. Alibali M. W. Nathan M. J. (2008). Trade-offs between grounded and abstract representations: evidence from algebra problem solving. Cogn. Sci. 32, 366–397. doi: 10.1080/03640210701863933, PMID: 21635340

[ref24] Lachner A. Nückles M. (2015). Bothered by abstractness or engaged by cohesion? Experts’ explanations enhance novices’ deep-learning. J. Exp. Psychol. Appl. 21, 101–115. doi: 10.1037/xap0000038 25437792

[ref25] Lamnina M. Chase C. C. (2019). Developing a thirst for knowledge: how uncertainty in the classroom influences curiosity, affect, learning, and transfer. Contemp. Educ. Psychol. 59:101785. doi: 10.1016/j.cedpsych.2019.101785

[ref26] Littell R. C. Milliken G. A. Stroup W. W. Wolfinger R. D. Schabenberger O.. (2006). SAS for mixed models, 2nd ed. Cary, NC: SAS Institute Inc.

[ref27] Lopez E. J. Shavelson R. J. Nandagopal K. Szu E. Penn J. (2014). Ethnically diverse students' knowledge structures in first-semester organic chemistry. J. Res. Sci. Teach. 51, 741–758. doi: 10.1002/tea.21160

[ref28] Markman A. B. Gentner D. (1996). Commonalities and differences in similarity comparisons. Mem. Cogn. 24, 235–249. doi: 10.3758/BF03200884 8881326

[ref29] Mayer R. E. (2004). Should there be a three-strikes rule against pure discovery learning? Am. Psychol. 59, 14–19. doi: 10.1037/0003-066X.59.1.14, PMID: 14736316

[ref30] Nokes-Malach T. J. VanLehn K. Belenky D. M. Lichtenstein M. Cox G. (2012). Coordinating principles and examples through analogy and self-explanation. Eur. J. Psychol. Educ. 28, 1237–1263. doi: 10.1007/s10212-012-0164-z

[ref31] Novick L. R. (1988). Analogical transfer, problem similarity, and expertise. J. Exp. Psychol. Learn. Mem. Cogn. 14, 510–520. doi: 10.1037/0278-7393.14.3.510, PMID: 2969945

[ref32] Novick L. R. Holyoak K. J. (1991). Mathematical problem solving by analogy. J. Exp. Psychol. Learn. Mem. Cogn. 17, 398–415. doi: 10.1037/0278-7393.17.3.398, PMID: 1829473

[ref33] Rattermann M. J. Gentner D. (1998). More evidence for a relational shift in the development of analogy: children's performance on a causal-mapping task. Cogn. Dev. 13, 453–478. doi: 10.1016/s0885-2014(98)90003-x

[ref34] Reed S. K. (1989). Constraints on the abstraction of solutions. J. Educ. Psychol. 81, 532–540. doi: 10.1037/0022-0663.81.4.532

[ref35] Reed S. K. Bolstad C. A. (1991). Use of examples and procedures in problem solving. J. Exp. Psychol. Learn. Mem. Cogn. 17, 753–766. doi: 10.1037/0278-7393.17.4.753

[ref36] Reed S. K. Stebick S. Comey B. Carroll D. (2012). Finding similarities and differences in the solutions of word problems. J. Educ. Psychol. 104, 636–646. doi: 10.1037/a0027181

[ref37] Renkl A. (1997). Learning from worked-out examples: a study on individual differences. Cogn. Sci. 21, 1–29. doi: 10.1207/s15516709cog2101_1

[ref38] Revelle W. R. (2019). Psych: Procedures for personality and psychological research.

[ref39] Richland L. E. McDonough I. M. (2010). Learning by analogy: discriminating between potential analogs. Contemp. Educ. Psychol. 35, 28–43. doi: 10.1016/j.cedpsych.2009.09.001

[ref40] Richland L. E. Simms N. (2015). Analogy, higher order thinking, and education. Cogn. Sci. 6, 177–192. doi: 10.1002/wcs.1336, PMID: 26263071

[ref41] Rittle-Johnson B. Alibali M. W. (1999). Conceptual and procedural knowledge of mathematics: does one lead to the other? J. Educ. Psychol. 91, 175–189. doi: 10.1037/0022-0663.91.1.175

[ref42] Rittle-Johnson B. Star J. R. (2007). Does comparing solution methods facilitate conceptual and procedural knowledge? An experimental study on learning to solve equations. J. Educ. Psychol. 99, 561–574. doi: 10.1037/0022-0663.99.3.561

[ref43] Ross B. H. Kennedy P. T. (1990). Generalizing from the use of earlier examples in problem solving. J. Exp. Psychol. Learn. Mem. Cogn. 16, 42–55. doi: 10.1037/0278-7393.16.1.42

[ref44] Ross B. H. Kilbane M. C. (1997). Effects of principle explanation and superficial similarity on analogical mapping in problem solving. J. Exp. Psychol. Learn. Mem. Cogn. 23, 427–440. doi: 10.1037/0278-7393.23.2.427

[ref45] Schwartz D. L. Chase C. C. Oppezzo M. A. Chin D. B. (2011). Practicing versus inventing with contrasting cases: the effects of telling first on learning and transfer. J. Educ. Psychol. 103, 759–775. doi: 10.1037/a0025140

[ref46] Sloutsky V. M. Kaminski J. A. Heckler A. F. (2005). The advantage of simple symbols for learning and transfer. Psychon. Bull. Rev. 12, 508–513. doi: 10.3758/BF03193796, PMID: 16235637

