# Supplementary material for: Mapping principles and worked examples for structural learning: effects of content complexity
Source: Front Psychol. 2023 Aug 23;14:1241873. doi: 10.3389/fpsyg.2023.1241873 (PMC10481337; doi:10.3389/fpsyg.2023.1241873)
Supplement: Supplementary file 1 [file Data_Sheet_1.PDF]

Supplemental Materials

Mapping Principles and Worked Examples for Structural Learning: Effects of Content Complexity

Hsinmei Liao*

***Correspondence:** Hsinmei Liao: hliao@mail.tcu.edu.tw

1 Sample Size Estimation

The information on effect sizes suitable for the purposes of the study was scant in the literature. Therefore, an arbitrary criterion was used to determine the possible effect size. It was hoped that the sample size was able to allow the study to detect a relatively small but meaningful size of difference between the conditions. To this end, an effect size *d* of value 0.4 (close to the medium values) was used to estimate the sample size for this study.

The standard deviation used to calculate the sample size was determined using the method (assuming normal distribution) discussed in Ryan (2013; Chapter 2). A more conservative value of *SD* = 2 was used so that the experiment might have enough power when there were greater variances in the measures. A hypothetical dataset of group means was constructed for estimating the sample size according to the above discussion. There were two means for each of the five conditions, one for each principle. Note that because it was unlikely to generate means that yield an effect size of 0.4 for all interested comparisons, the effect size used for estimating the sample size was in fact between 0.35 and 0.40. (A smaller *d* requires more samples which will not decrease the power of the study.) The means for the conditions on the simple and complex principle respectively were as the following. For demonstration, the effect size for the PEs versus PEc condition on the simple principle was (6.3 - 5.6) /2 = 0.35; for the EsEs versus EsEc condition was (7.2 – 6.4) / 2 = 0.4.

PEs	6.300	5.500
PEc	5.600	4.800
EsEs	7.200	6.400
EsEc	6.400	5.600
EcEc	6.500	5.700

Furthermore, no information was available on the correlation between the two principles on any dependent measures. Therefore, analysis was conducted using correlation of small to medium sizes 0.3 and 0.5. The model for estimating the sample size had the condition variable (i.e. the five conditions) as the between-subject factor and the principle variable as the within-subject factor. For power of 0.80 and Type I error of .05, the SAS GLMPOWER yielded a size of 125 and 145, respectively, for correlation of 0.5 and 0.3 on the basis of the means of the two

dependent measures. To balance between the sources of the study and the chance of detecting the effects, the final sample size was set at about 145. A set of sample SAS codes is presented below.

```
proc glmpower;
  class map;
  model geomu poimu=map;
  repeated principle;
power
        effects=(map)
        mtest=hlt
        alpha=0.05
        power=0.8
        ntotal=.
        stddev=2.00
        matrix ("DVcorr")=(1.0000
                    0.30 1.0000)
        corrmat="DVcorr";
title 'effect size=0.35-0.40, data: sd=2, r=0.3';
run;
```

2 Determination of the Response Patterns on the Multiple-Choice Task

For each principle, the item difficulties of the task were determined by the mean number of correct responses on the items and the test results of all pair of items. A response pattern for each participant was then constructed by arranging the items from the easiest to the most difficult. A normal pattern should be one that if the more difficult items were answered correctly then the easier ones should also be answered correctly.

Take the simple principle as an example, the item means from the highest to the lowest were item1, item5, item4, item2, item3. Thus, a response pattern was determined by the responses on

the five items in this order. According to the pairwise comparisons of these items, controlling Type 1 error at 0.05, the following were not significantly different: item1 and item5, item5 and item4, item4 and item2, and finally item2 and item3. Therefore, the normal response patterns included the following:

00000,

01000,

10000,

10100,

11000,

11010,

11011,

11100,

11101,

11110,

11111.

3 References

Ryan, Thomas P. 2013. *Sample size determination and power*. US: John Wiley & Sons.
