# Supplementary material for: Mapping principles and worked examples for structural learning: effects of content complexity
Source: Front Psychol. 2023 Aug 23;14:1241873. doi: 10.3389/fpsyg.2023.1241873 (PMC10481337; doi:10.3389/fpsyg.2023.1241873)
Supplement: Supplementary file 2 [file Data_Sheet_2.pdf]

Appendix

Exemplary Experimental Materials

1. Exemplary Content from the Prerequisite-Study booklet

Probability Functions and Distributions

Suppose a random variable X has random values $x_1, \ldots, x_n$. There is only a probability corresponding to an x_i, and the probability is represented as $f(X = x_i)$ *or* $f(x_i)$, which also satisfies the following two requirements:

(1) $0 \le f(x_i) \le 1$

(2) $\sum_{i=1}^{n} f(x_i) = 1$

Thus, $f(X)$ is the probability function, or probability distribution, for X that is the distribution of the probability of the random variable occurring in each random value.

Expected Value

An expected value, also called a mean, is the value or outcome expected to occur or be observed if we repeat an experiment many times.

2. The PEs Pair for the Simple Principle in the Work Booklet

Assume conducting a series of independent trials until the first success. On every trial, the probability of success is p and the probability of failure is $1 - p$, $0 < p < 1$. Let X be the number of trials needed, then X is a geometric random variable. Its probability distribution is

$$f(x) = (1-p)^{x-1} p \quad x = 1, 2, 3, \ldots$$

According to statistics, when a motorcycle traverses an intersection, the probability of having an accident is 0.01, and the probability of passing through safely is 0.99. A recording is

taken at an intersection starting from midnight. What is the probability that all the motorcycles passed through safely but the 20th had an accident?

$$f(20) = 0.99^{20-1} \times 0.01 \ = 0.99^{19} \times 0.01 = 0.8262 \times 0.01 = 0.0083$$

The probability is 0.0083 ◦

3. The PEc Pair for the Complex Principle in the Work Booklet

If an event satisfies the following three conditions, it is called a Poisson random experiment.

(1) The number of events occurring in a continuous space is independent of the number in another space.

(2) The expected value of events occurring in a continuous space is proportional to the size of the space.

(3) In a very little space, event will occur once or not.

Assume in a certain space the expected value of event A occurring is λ. Let X be the number of events occurred in the space, then X is a Poisson random variable. Its probability distribution is

$$f(x) = \frac{\lambda^x e^{-\lambda}}{x!} \qquad x = 0,\ 1,\ 2,\ 3,\ \ldots,\ \infty \qquad e = 2.718281828$$

The reception desk at a certain school received an average of 3 calls in an hour. What is the probability of receiving the number of phone calls that is one third of the expected value in two hours?

$$3 \times 2 = 6$$
$$6 \div 3 = 2$$
$$f(2) = \frac{6^2 \times 2.718281828^{-6}}{2!} = \frac{36 \times 0.0025}{2 \times 1} = \frac{0.0892}{2} = 0.0446$$

The probability is 0.0446 ◦

4. Free Mapping Task in the Work Booklet for the Simple Principle

(1) In terms of relevance, what are the commonalities between Example 1 and the geometric distribution? Please write down every one of them.

(2) (continue from the previous question) What are the differences between Example 1 and the geometric distribution? Please write down every one of them.

5. Guided Mapping Task for the Simple Principle in the Work Booklet for the PE Conditions

According to the geometric distribution, combine the following terms in the most meaningful ways. First of all, write down the complete sentence with the combination of the terms. For example, “success” and “probability *p*” can be combined into “the probability of success is *p*.” Next, find out from Example 1 the part that corresponds to the sentence. For example, “the probability of having an accident is 0.01” in Example 1 corresponds to “the probability of success is *p*.”

- failure	- one time	- between 0 and 1
- between 1 and infinity	- x trials	- $f(x)$
- probability p	- probability $1 - p$	- the first success
- independent trial	- a sequence of	- success
